# Predicting the Effect of Bilateral Pelvic Osteotomy on Sagittal Alignment Correction and Surrounding Muscles: A Mathematical Model

**DOI:** 10.1155/2019/3041359

**Published:** 2019-02-03

**Authors:** Vahhab Zarei, Sharon C. Yson, Joan E. Bechtold, Jonathan N. Sembrano

**Affiliations:** ^1^PhD Student, University of Minnesota, 2450 Riverside Ave. S R200, Minneapolis, MN 55454, USA; ^2^Spine Research Associate, University of Minnesota, 2450 Riverside Ave. S R200, Minneapolis, MN 55454, USA; ^3^Vice Chair, Research, Gustilo Professor of Orthopaedic Research, Department of Orthopedic Surgery, University of Minnesota, 2450 Riverside Ave. S R200, Minneapolis, MN 55454, USA; ^4^Associate Professor, Department of Orthopaedics, University of Minnesota, 2450 Riverside Ave. S R200, Minneapolis, MN 55454, USA

## Abstract

*Study Design. *Mathematical Model.* Objectives. *To investigate the relationship between pelvic osteotomy opening angle (OA) and its effect on spinopelvic sagittal parameters as well as the resting length of surrounding muscles.* Methods. *Predictive equations correlating OA with spinopelvic parameters were derived using geometric relationships. A geometric model calculated spinopelvic parameters (SVA, pelvic incidence [PI], PT, and T1 pelvic angle [TPA]) produced by progressively increasing the OA. These values were compared to optimal balance criteria in the literature. Four muscles crossing the osteotomy site were evaluated: Gluteus Medius (GMED), Gluteus Maximus (GMAX), Piriformis (P), and Tensor Fascia Lata (TFL). Insertion points were obtained from an OpenSim software model. GMAX and GMED were subdivided into 3 (anterior, middle, and posterior).* Results. *OA correlated negatively with PI, TPA, and SVA and positively with PT. From baseline SVA of 22 cm, OA 21° reduced SVA to 5cm. OA 23° reduced TPA to 14°. OA 30° increased PT to 20°. OA 26° decreased PI-LL to 10°. OA range of 26°-30° resulted in optimal sagittal deformity correction. OA correlated with SR positively for TFL and anterior GMED and negatively for the rest of muscles. For this OA, the SR approximately decreased 6%, 5%, 6%, 8%, and 5% for posterior GMED, anterior GMAX, middle GMAX, posterior GMAX, and P, respectively. It increased 8% and 4% for anterior GMED and TFL, respectively.* Conclusion. *Predictive relationships between osteotomy OA and spinopelvic parameters were shown, providing proof of concept that sagittal balance may be achieved via pelvic osteotomy.

## 1. Introduction

Surgeons have known for many years that a flattened lumbar lordosis (flatback syndrome) has adverse effects on surgical outcome. Lazennec et al. (2000) recognized that fusion should not only be the main goal of lumbar spine surgery; they have shown that failure to restore normal sagittal alignment correlated with postfusion pain [1]. However, it was not until the mid-2000s when Glassman et al. demonstrated clear statistical correlation between sagittal alignment and outcomes as measured by validated tools that primacy was placed on restoring and/or preserving sagittal alignment when performing spinal fusion surgery [2]. Even in patients who present with coronal deformity (i.e., scoliosis), sagittal alignment was still shown to be significantly more predictive of clinical outcome compared to coronal deformity correction [3].

Since then, Schwab et al. have formulated objective radiographic goals for surgeons to aim for when performing these surgeries. Among these are the approximation of pelvic incidence (PI) and lumbar lordosis (LL) (PI – LL < 9°), with reduction of compensatory posterior pelvic tilt (PT < 20°) and neutralization of sagittal vertical axis (SVA < 5 cm) [4]. More recently, Protopsaltis et al. introduced the T1 pelvic angle (TPA) and proposed an additional radiographic measurement goal (TPA < 14°) [5]. In the past few years, spine surgeons have been more commonly requesting for full-spine standing (lateral) radiographs, both for preoperative planning and for postoperative evaluation, with inclusion of the femoral heads, to facilitate measurement of the aforementioned sagittal parameters.

An adverse consequence of increased emphasis on sagittal alignment correction has been the concomitant increase in multilevel fusion surgery and complex osteotomies (e.g., pedicle subtraction osteotomy), with resultant increased cost and morbidity. Over a span of a decade (2000-2010), the average charge per inpatient admission increased by approximately 230% and 190% for Medicare and private insurance, respectively [6]. Surgery for adult spinal deformities is technically demanding and has high complication rates [7, 8].

**Figure 1:**
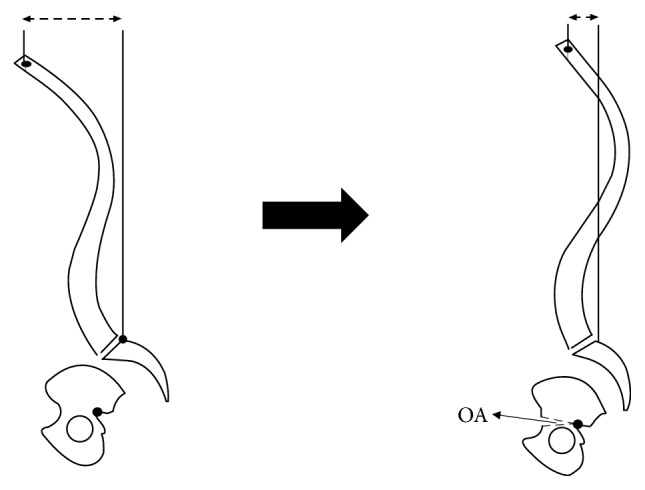
Schematic representation of the sagittal view of the spinopelvis system showing proposed bilateral pelvic osteotomy (BPO) procedure. Osteotomy will be performed caudal to SIJ but above the hip joints. A small change in pelvis opening angle (OA) will result in considerable sagittal vertical axis (SVA) correction.

We propose a potential alternative to spinal osteotomies for spinal realignment surgery by creating the fulcrum of rotational correction more caudally at the pelvis, thus providing a longer lever arm to bring the head back in a balanced position. In other words, a greater change to the sagittal vertical axis could be produced by a relatively smaller angular change at the pelvic osteotomy site. To avoid injuries to the thecal sac and nerve roots within the spinal canal as well as to the sacroiliac joints (SIJ), we propose that the osteotomy be performed caudal to the SIJ but above the hip joints. These would be bilateral opening wedge osteotomies (BPO) from the anterior inferior iliac spine (AIIS) to the sciatic notch on each side of the pelvis. The posterior cortex along the sciatic notch will serve as the fulcrum of rotation in the sagittal plane.  Figure 1 schematically shows how this osteotomy will be performed.

The objective of this study was to evaluate the capability of our proposed pelvic osteotomy to correct spinal sagittal malalignment using a mathematical predictive model. Osteotomy opening angle effects on sagittal spinopelvic parameters as well as resting length of muscles crossing the osteotomy site were examined. This study lays the groundwork for stepwise implementation of BPO for sagittal plane realignment in human patients.

## 2. Methods

### 2.1. Part A: Assessment of Sagittal Alignment after Virtual Bilateral Pelvic Opening Wedge Osteotomies

**Figure 2:**
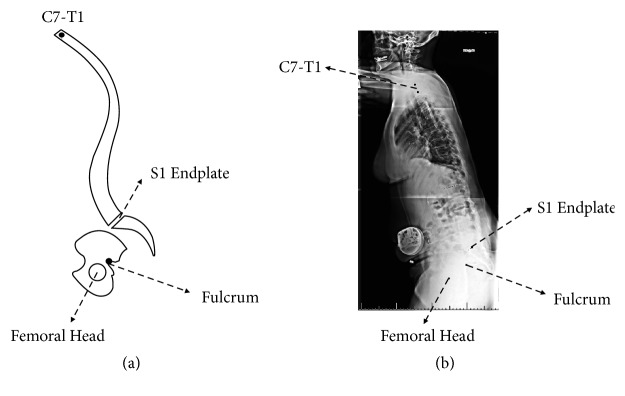
Diagram of the sagittal view of the spinopelvic system showing landmark points used to measure angles and distances used in the predictive equations (a). The location of these points was identified and measured in an actual full sagittal spine X-ray (b).

To evaluate the effect of BPO on sagittal alignment, predictive equations correlating pelvis opening angle (OA) with spinopelvic parameters were derived. These equations are based on anatomic landmarks (femoral head, BPO fulcrum, S1 endplate, and C7-T1 vertebral bodies) as shown in Figure 2(a). To demonstrate feasibility, a lateral full-spine X-ray of one patient with sagittal deformity (Figure 2(b)) was used identify the location of these points. Spinal curvature was assumed to remain fixed (i.e., to represent rigid spinal deformity). Predictive equations correlating OA with spinopelvic parameters were derived using trigonometric-geometric relationships:

(1) SVA=Lsin⁡T1SPA+L′cos⁡α4−OATPA=T1SPA−α5−β+π2PI=sin−1⁡sin⁡α2+OA1+k2−2kcos⁡α2+OA+α1PT=PI+π2−α1−α3+OA

where

(2) β=sin−1sin⁡α2+OA1+k′2−2k′cos⁡α2+OA⁡T1SPA=sin−1sin⁡α6+OA1+k′′2−2k′′cos⁡α6+OA+α5−π2

And

(3) k=bak′=abk′′=aL

**Figure 3:**
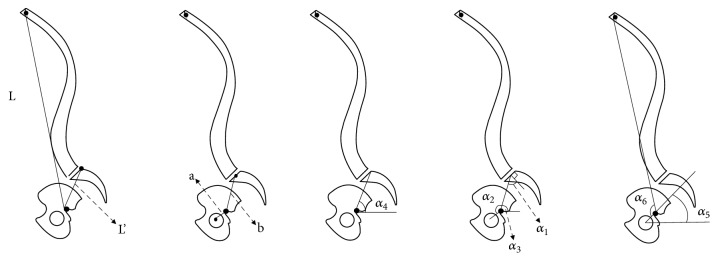
Lateral spine diagram showing angles used in the predictive equation.

These equations are expressed in terms of OA, as well as patient-specific distances and angles (Figure 3). These lengths and angles can be easily calculated based on landmark points shown in Figure 2.

Changes of SVA, TPA, PT, and PI-LL with OA were plotted in figures based of which the acceptable range of OA can be specified. The allowable OA was confined to satisfy these conditions:

(4) −50mm<SVA<50mmTPA<14°PT<20°PI−LL<9°

### 2.2. Part B: Effect of Osteotomy on Involved Muscles

Aside from spinopelvic parameters, muscles passing across the osteotomy site are also under influence of BPO, as their resting length change because of the relative changes in their insertion points. Four muscles which cross the osteotomy site were studied: gluteus maximus (GMax), gluteus medius (GMed), tensor fasciae latae (TFL), and piriformis (P).

**Figure 4:**
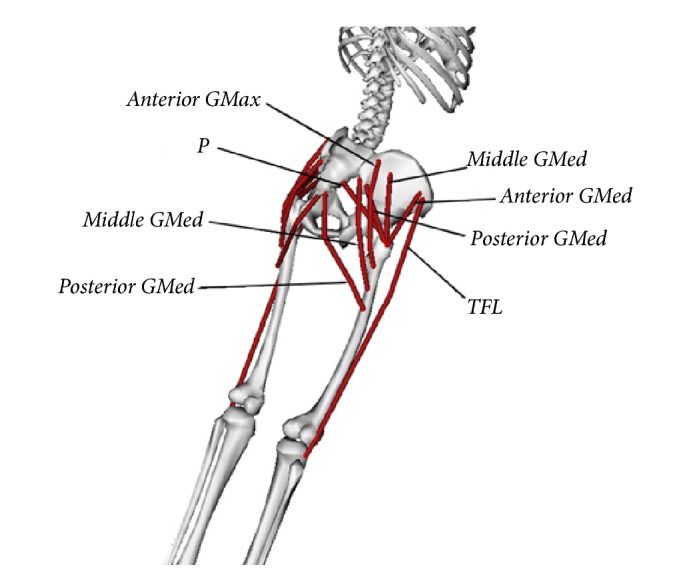
Schematic diagram of surrounding pelvic and hip muscles evaluated in the proposed procedure [9].

™GMax and GMed are relatively wide muscles and they may undergo shortening or lengthening depending on location over their span. Thus, each of them was subdivided into three sections (named anterior, posterior, and middle), each assigned a uniform initial length (Figure 4). All of the muscle segments are considered as one dimensional longitudinal part.

A predictive equation was derived based on basic trigonometric laws to postoperatively estimate the length of each muscle:

(5) $$\begin{array}{c} d=\sqrt{l^{2}+{\Delta z}^{2}} \end{array}$$

where *d* is the muscle length and:

(6) $$\begin{array}{c} l=\sqrt{l_{1}^{2}+l_{2}^{2}-2l_{1}l_{2}\cos\left(\beta +OA\right)} \end{array}$$

**Figure 5:**
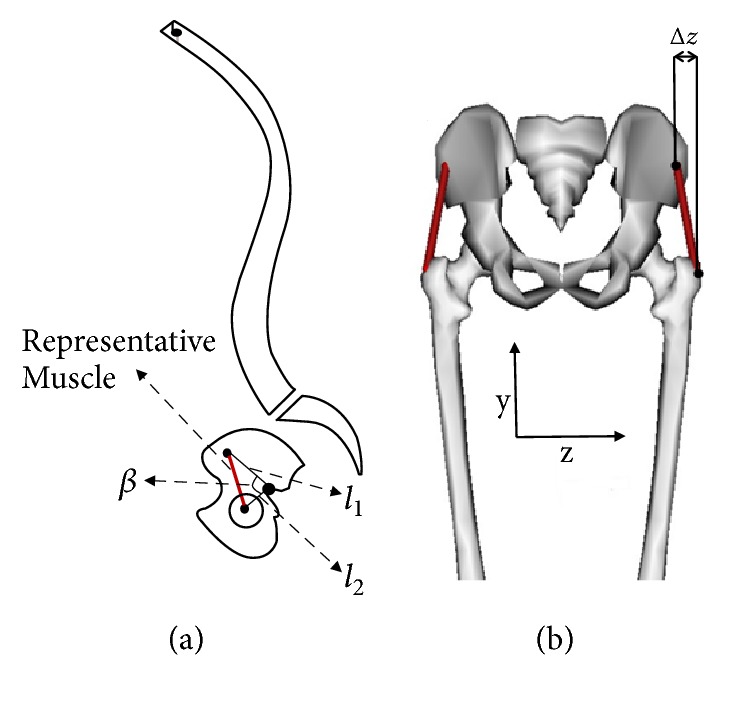
Sagittal Representation of the spine (a) and posterior representation of the pelvis and hip (b) showing lengths l_1, l_2, Δz, and angle *β* used in the predictive equation for muscle length. These parameters are different for each muscle segment. The red line represents a muscle segment [9].

*l*_1_, *l*_2_, Δ*z*, and angle *β* in the equations are shown in Figure 5, with the z axis defined perpendicular to the sagittal plane. Since these parameters vary for different subjects and it is not possible to quantify them with spine X-ray images, OpenSim [9] software was utilized. OpenSim is an open source muscle modeling software from which muscle segment lengths were estimated.

Muscle-stretch ratio (SR) is defined as the ratio of new muscle length over initial length where *L*_0_ is initial length and *λ* is the stretch ratio:

(7) $$\begin{array}{c} \lambda =\frac{d}{L_{0}} \end{array}$$

Finally, this stretch ratio for each muscle was plotted with OA, to check if it exceeds the critical stretch ratio in the literature (25%). Shortening muscles, that is muscle whose insertion points become closer after surgery, are assumed to be safe, as they do not generate any stress.

## 3. Results

**Figure 6:**
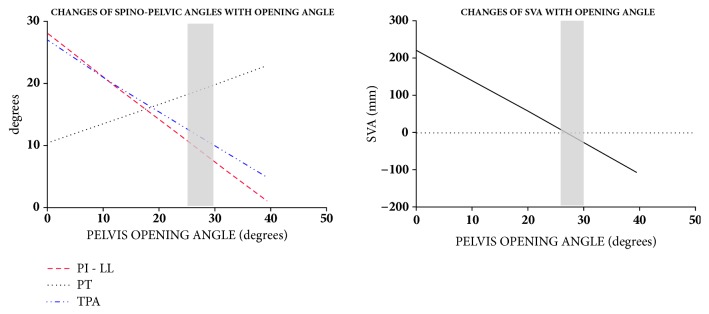
Graphs showing the relationship of osteotomy opening angle (OA) with spinopelvic parameters and SVA. The grey area represents the range of opening angles where optimal sagittal deformity correction is achieved.

The linear relationships between osteotomy opening angle (OA) and selected sagittal spinopelvic radiographic parameters are shown on Figure 6. OA correlated negatively with PI, TPA, and SVA and positively with PT. From baseline SVA of 22 cm, a 21° OA reduced SVA to 5 cm. A 23° OA reduced TPA to 14°. A 30° OA increased PT to 20°. A 26° OA decreased PI-LL to 10°. Thus, an OA range of 26° to 30° resulted in optimal sagittal deformity correction. Within this range, PI decreased by 19°-21° from baseline.

**Figure 7:**
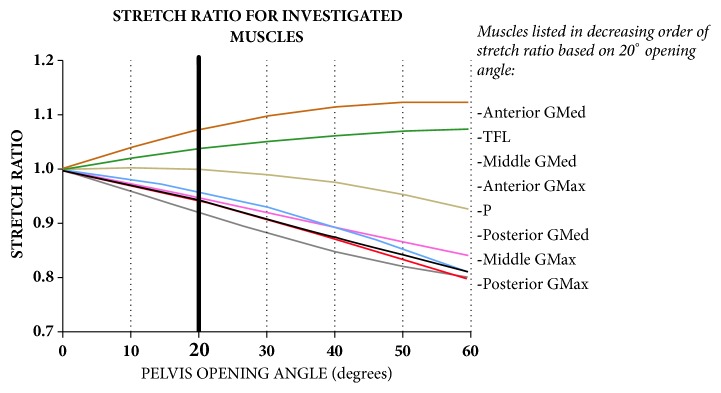
Graph showing the correlation of pelvis opening angle (OA) and the stretch ratio (SR) of selected muscle segments.

As can be seen in Figure 6, the nonlinear relations for spinopelvic parameters, as shown as predictive equations, yield to linear correlations for the practical range of opening angles. For the specific patient examined in this paper, the alignment parameters have the following linear correlations:

(8) SVA=−8.3OA+223.8PI=−0.7OA+54.4PT=0.3OA+10.4TPA=−0.6OA+27.0

where SVA is in mm and OA, PI, PT, and TPA are in degrees. OA correlation with muscle SR is shown in Figure 7. OA correlated positively with muscle SR for the tensor fascia latae (TFL) and the anterior portion of the gluteus medius (anterior GMed); it correlated negatively with SR for the other muscles, including the anterior, middle, and posterior portions of the gluteus maximus (anterior, middle, and posterior GMax), the middle and posterior portions of the gluteus medius (middle and posterior GMED), and the piriformis (P). At a preselected 20° OA, muscle SR decreased by 6%, 5%, 6%, 8%, and 5% for posterior GMed, anterior GMax, middle GMax, posterior GMax, and P, respectively, and increased by 8% and 4% for anterior GMed and TFL, respectively. There was no SR change for middle GMed.

## 4. Discussion

In this study we primarily sought to examine the feasibility of a proposed pelvic osteotomy procedure in producing effective spinal sagittal alignment change via geometric mathematical modeling. Specifically, we looked at the relationships between pelvic osteotomy opening angle and commonly used spinopelvic radiographic parameters and calculated the amount of opening angle necessary to bring these parameters within the range of what is considered acceptable in the literature. Secondarily, the relationships between osteotomy opening angle and resting length of muscles spanning the osteotomy site, as characterized by the muscle-stretch ratio, was examined.

Predictive relationships between pelvic osteotomy OA and spinopelvic parameters were shown in our mathematical model, thus providing proof of concept that sagittal alignment may be achieved via pelvic osteotomy. Because of the negative correlation between osteotomy opening angle and PI, TPA and SVA, a minimum OA can be defined based on the lowest OA that would still correct these 3 parameters to acceptable levels. On the other hand, because of the positive correlation between osteotomy opening angle and PT, a maximum OA can be defined based on the highest OA beyond which PT would likely become unacceptable. Thus an acceptable or target range of OA can be deduced from the model. In the example used for the study, the minimum and maximum OA were 26° and 30°, respectively, thus providing a 4° range of acceptable OA. It should be pointed out, however, that these values were arrived at by modeling based on a single patient's radiograph. Thus, the ideal 26-30 degree range of opening angle is specific only to this patient. Given the wide spectrum of deformity, each patient would have his/her own range of ideal opening wedge angle.

There are other limitations to our study that should be discussed. (1) While we looked at the potential effects of bilateral pelvic osteotomies on spinal sagittal parameters, we have not performed such a procedure on actual patients for sagittal deformity correction. Thus, we are unable to make specific recommendations on aspects such as fixation method, type of bone graft, need for postoperative immobilization, and weight-bearing status. We speculate that the posterior iliac cortex should be preserved in order to maintain stability, that fixation has adequate rigidity to allow bed to chair transfers and ideally even weight-bearing, and that the bone graft should also have strong structural properties for supplemental stability. These will be the focus of future studies. (2) The assumption of a rigid spine may not be true for most cases of sagittal deformity. This may, however, hold true in cases where previous multilevel fusion was performed, whether the fusion was (a) the cause of the deformity (‘iatrogenic flatback') or (b) performed for the deformity but inadequate correction was achieved.

There are important differences between our pelvic osteotomy procedure and conventional spinal osteotomies in terms of their effects on individual spinopelvic parameters and how spinal alignment is achieved. In conventional spine deformity surgery, correction is mainly effected on lumbar lordosis (LL), whereas pelvic incidence (PI) is regarded as a fixed parameter [10–12]. By performing the osteotomy in the pelvis, however, the fixed relationship between the hip joint and the sacrum is disrupted, and correction is effected instead on pelvic incidence. In this scenario, the lumbar lordosis is presumed to be a fixed parameter, such as in cases with prior multilevel fusion. With either method, matching between PI and LL is ultimately accomplished, either by increasing LL (traditional spinal osteotomy) or by decreasing PI (pelvic osteotomy).

Recently, Bodin and Roussouly from France published their case series on the use of Salter type osteotomy and sacral pedicle subtraction osteotomy to correct sagittal spine deformities [13]. The majority of their cases were diagnosed with spondyloptosis. Prior to surgery on actual patients, they performed mathematical modeling by doing virtual osteotomies on pelvic x-rays to predict the effect of the procedure on PI. They reported that, with Salter osteotomy, there is an inverse correlation between opening angle and PI. The same trend can be seen with our model. However, in addition to PI change, we also evaluated the effect of the osteotomy opening angle on SVA, TPA, and PT. Perhaps the foremost difference between the two models is that Bodin and Roussouly's was derived by performing virtual osteotomies on X-ray images and our predictive equations were based on fundamental geometric principles.

Another recent case study discussed the use of posterior sacral osteotomies to correct a fixed iatrogenic spinopelvic deformity. The technique involved longitudinal osteotomies through the sacral ala and derotation of the sacrum to achieve the desired pelvic incidence. At one year after surgery, the patient reportedly maintained a balanced sagittal alignment [14].

While the osteotomy we propose is in many respects similar to the Salter innominate osteotomy, it is fundamentally different in both its goal and the mechanism by which it is achieved. As with Salter, a linear osteotomy is created from the anterior surface of the ilium above the acetabulum towards the sciatic notch. An anterior opening wedge is then created with the fulcrum along the sciatic notch. The Salter osteotomy is meant to provide better coverage for the femoral head by redirecting the acetabulum; this is achieved by creating a pivot point at the pubic symphysis in addition to the posterior fulcrum. Thus, in order for this osteotomy to achieve the desired effect, it is by definition a unilateral osteotomy (even though subsequent authors have reported on bilateral Salter osteotomies). On the other hand, our pelvic osteotomy has the goal of correcting sagittal deformity by retroverting the superior portion of the pelvis. Thus, by definition this has to be performed bilaterally, and the correction achieved simultaneously.

We acknowledge that spinal osteotomies are effective and well-established procedures in the spine surgeon's armamentarium. Our conceptualized bilateral pelvic osteotomy is not meant to supplant these more traditional procedures, but rather to present it as a possible alternative in certain situations. These may include (1) previous multilevel fusion as mentioned above; (2) ankylosed spine in absence of previous surgery (e.g., ankylosing spondylitis); and (3) cases of abnormally high pelvic incidence (~ > 75 degrees) where creating lumbar hyperlordosis to achieve PI-LL matching may not be deemed desirable because of potential concerns with symptomatic stenosis and facet degeneration. Lastly, if future studies are able to show that a pelvic osteotomy procedure is a smaller physiological hit to the body compared to spinal 3-column osteotomy surgery, patients with poor medical status who cannot tolerate the latter may be considered as candidates for pelvic osteotomy instead.

Whether planning for sagittal correction via spinal osteotomy or pelvic osteotomy, it is important to note that a posteriorly tilted pelvis (high PT) causes partial correction of the SVA. Therefore, the whole image has to be rotated anteriorly to ‘correct' PT prior to planning the osteotomy; this step unmasks the true extent of the deformity without the mitigating effects of posterior pelvic tilt. Failure to rotate the image results in under-correction of the deformity.

An important limitation of our mathematical model is in terms of predicting final pelvic tilt (PT) and sagittal vertical axis (SVA). Unlike PI, LL, and TPA, PT and SVA are significantly affected by the position of the hip joint in the sagittal axis. Posterior rotation or extension of the hip joint produces posterior pelvic tilt or retroversion, a primary compensatory mechanism in the presence of sagittal deformity. By correcting the deformity, it is presumed that there will be concomitant normalization of PT. However, unlike LL (in spinal osteotomy) or PI (in pelvic osteotomy) this is not directly locked in position during surgery. Potential factors that may prevent PT normalization despite deformity correction may include hip extension contracture, hip flexion weakness and hip joint stiffness.

Because all the muscles that cross the pelvic osteotomy site also cross and help position the hip joints, we also examined the relationship between osteotomy opening angle and resting length of selected muscles, expressed as the individual muscle-stretch ratio (SR). Our model showed relationships between OA and individual muscle SR. In general, muscles that cross the osteotomy site anterior to the fulcrum of rotation at the osteotomy site tend to lengthen with opening of the osteotomy (tensor fascia latae and anterior gluteus medius), and posterior muscles tend to shorten (anterior, middle and posterior gluteus maximus, posterior gluteus medius and piriformis). Because the gluteus medius and maximus muscles have very broad muscle bellies, they were likely to experience differential lengthening or shortening of their fibers at different points in the muscles; thus, these muscles were divided each into anterior, middle and posterior portions. It is worth noting that because the gluteus medius spans the length of the osteotomy, the anterior portion of the muscle lengthens, the middle portion stay relatively isometric, and the posterior portion shortens with increasing opening of the osteotomy. Despite our ability to show effects of osteotomy angle on muscle SR, we cannot say whether there would be a measurable or clinically relevant effect on muscle function and body posture. It is, however, unlikely that pelvic osteotomy within the calculated recommended range to correct spinal alignment will adversely affect muscle function since our maximum SR (8% for anterior GMed) is substantially less than the reported critical SR value (25.4%) beyond which muscle function is significantly affected in animal models [15]. Lastly, this model does not take into account muscle effects of iatrogenic injury during surgery and of postoperative physical therapy.

A pelvic osteotomy procedure may also have unintended consequences on the hip joint(s); this may either be in terms of (a) position of the hip (cup ante/retroversion) or (b) potential acetabular surface deformation given osteotomy's proximity to the acetabulum. The former may lead to increased predisposition to hip instability/dislocation, particularly in the presence of total hip arthroplasty. This was touched upon by a recent publication showing decreased cup anteversion (which may lead to increased risk of prosthetic hip dislocation) after spinal deformity correction [16]. Whether the latter (b) issue will become clinically manifest is unknown. While there are pelvic osteotomies developed and currently utilized to mold or reconfigure the acetabulum for hip dysplasia, these are only performed in the very young patient population; it is unlikely that such reconfiguration also occurs in adult hips, although violation and subsequent weakening of the periacetabular subchondral bone is a possibility and cause of concern.

For mathematical modeling purposes, it was assumed that the spine is completely rigid. While this may hold true for clinical conditions such as ankylosing spondylitis, advanced multilevel spondylosis with autofusion and, perhaps more commonly, patients who have had extensive fusion surgery, it is much more difficult to predict how a mobile/flexible spine would respond to bilateral anterior opening wedge pelvic osteotomies. We hypothesize that there may still potentially be a role for such an osteotomy in patients who have an abnormally high pelvic incidence (~ > 75°) and preferably prior to onset of degenerative changes at the lower lumbar levels secondary to chronic compensatory hyperlordosis. While we intuitively suspect that an osteotomy aimed at decreasing the pelvic incidence would lead to concomitant decrease (relaxation) of lumbar hyperlordosis without leading to a negative change in sagittal vertical axis, this is not supported by our mathematical model, as it does not take into account spinal flexibility

In summary, we have evaluated the feasibility of our proposed bilateral anterior opening wedge pelvic osteotomy as an alternative to traditional spinal osteotomy procedure for sagittal plane deformity correction. Using a geometric mathematical model, we demonstrated direct relationships between pelvic osteotomy opening angle and commonly used spinopelvic radiographic parameters (negative correlation with PI, SVA, and TPA; positive correlation with PT). In our model, we were able to accomplish correction of spinopelvic parameters to within accepted goals, thus demonstrating proof of concept that spinal alignment may be achieved by pelvic osteotomy in the setting of sagittal deformity. Lastly, we demonstrated effects of osteotomy opening angle on resting muscle lengths (expressed by individual muscle-stretch ratio) of selected muscles crossing the osteotomy site and hip joint.

## Data Availability

The detailed mathematical computations used to support the findings of this study are available from the corresponding author upon request.
